# Uptake of evidence in policy development: the case of user fees for health care in public health facilities in Uganda

**DOI:** 10.1186/s12913-014-0639-5

**Published:** 2014-12-18

**Authors:** Juliet Nabyonga-Orem, Freddie Ssengooba, Rhona Mijumbi, Christine Kirunga Tashobya, Bruno Marchal, Bart Criel

**Affiliations:** WHO Regional Office for Africa, Health systems and services cluster, P.O Box 6, Brazaville, Congo; Makerere University, School of Public Health, P.O. Box 7072, Kampala, Uganda; Regional East African Community Health (REACH) policy initiative, Uganda, College of Health Sciences, P.O. Box 7072, Kampala, Uganda; Institute of Tropical Medicine Antwerp-Belgium, Nationalestraat 155, 2000 Antwerp, Belgium

**Keywords:** User fees, Health care, Public facilities, Policy development, Knowledge translation, Uganda

## Abstract

**Background:**

Several countries in Sub Saharan Africa have abolished user fees for health care but the extent to which such a policy decision is guided by evidence needs further exploration. We explored the barriers and facilitating factors to uptake of evidence in the process of user fee abolition in Uganda and how the context and stakeholders involved shaped the uptake of evidence. This study builds on previous work in Uganda that led to the development of a middle range theory (MRT) outlining the main facilitating factors for knowledge translation (KT). Application of the MRT to the case of abolition of user fees contributes to its refining.

**Methods:**

Employing a theory-driven inquiry and case study approach given the need for in-depth investigation, we reviewed documents and conducted interviews with 32 purposefully selected key informants. We assessed whether evidence was available, had or had not been considered in policy development and the reasons why and; assessed how the actors and the context shaped the uptake of evidence.

**Results:**

Symbolic, conceptual and instrumental uses of evidence were manifest. Different actors were influenced by different types of evidence. While technocrats in the ministry of health (MoH) relied on formal research, politicians relied on community complaints. The capacity of the MoH to lead the KT process was weak and the partnerships for KT were informal. The political window and alignment of the evidence with overall government discourse enhanced uptake of evidence. Stakeholders were divided, seemed to be polarized for various reasons and had varying levels of support and influence impacting the uptake of evidence.

**Conclusion:**

Evidence will be taken up in policy development in instances where the MoH leads the KT process, there are partnerships for KT in place, and the overall government policy and the political situation can be expected to play a role. Different actors will be influenced by different types of evidence and their level of support and influence will impact the uptake of evidence. In addition, the extent to which a policy issue is contested and, whether stakeholders share similar opinions and preferences will impact the uptake of evidence.

**Electronic supplementary material:**

The online version of this article (doi:10.1186/s12913-014-0639-5) contains supplementary material, which is available to authorized users.

## Background

User fees for health care have been debated for well over a decade. Though some researchers have argued that they improve the quality of care, and subsequently utilization [1,2], others have pointed out negative effects such as deterring access to care, failure to realize meaningful financial contributions, and lack of visible improvements in quality [3,4]. In an effort to improve access to health services, several low income countries (LICs) have abolished user fees [5,6], but the results in the medium- to long-term have been mixed. Some countries have reported increased utilization, whereas others have reported a reduction in utilization and deteriorating quality of health services [7,8]. Designing successful reforms is not easy, and questions as to why results are mixed continue to be explored. Some scholars have highlighted the need for; paying careful attention to the process of designing reforms, evidence-based decision making and obtaining comprehensive evidence prior to the design process [9,10]. The definition of what evidence is and how much of it is enough also continues to be debated with researchers and academics expressing preference for peer-reviewed research [11]. However, some researchers have stated that, in the case of developing countries, evidence is much broader, consisting of monitoring reports, experience, and know how [12]. Bowen Zwi et al. also defined evidence broadly to encompass research, opinion and views of individuals or groups, results of consultative processes and published reports and documents [13]. Lomas et al. on the other hand argues that evidence concerns facts which may be actual or asserted and these may be known through experience or observation[14]. Scholars have further emphasized the importance of integrating research evidence and other types of policy relevant evidence, especially that which is considered as evidence by policy makers and stakeholders, without prioritizing one over the other but as complimentary input into policy development[15-17]. In this paper, we define evidence broadly to encompass published and unpublished research, routine monitoring reports, community complaints, clinician observations, and population-based surveys.

The policy-making process is influenced by several factors evidence being just one of them. Political processes, economic considerations, institutions, cultural issues and societal values all impact on health policy development [18,19]. Furthermore, the role of stakeholders in KT has been highlighted. Stakeholders are defined as individuals or institutions which are affected by the policy change, directly influence it, or have an interest in the outcome even when not directly involved [20]. The roles they play, their level of influence, interactions among them and their interest in a given issue, do impact the uptake of evidence [21,22].

In the past, the uptake of evidence has been restricted to instances in which the evidence led directly to development of a policy, a strategy or a guideline, but scholars have shown different ways in which evidence can be used. Evidence may be used to support a position that is already taken (symbolic use), to inform discussions and debate about a topical issue (conceptual use), or used to actually create guidelines or change practice directly (instrumental use) [23,24]. In this article, we consider symbolic, conceptual, and instrumental use as forms of evidence uptake. Much has been published on the effects of user fees on health care, but there is a dearth of literature on how much evidence guides decision making in such a reform.

In this paper, we look at the uptake of evidence in policy development, referred to as knowledge translation (KT), with specific reference to the abolition of user fees for health care in public facilities in Uganda. We use the term KT as defined by the Canadian Institute of Health Services Research: “a dynamic and iterative process that includes synthesis, dissemination, exchange, and ethically sound application of knowledge to improve health, provide more effective health services and products, and strengthen the health care system” [25]. We define policies as decisions made by those with responsibility for a given policy arena [26].This study is part of a bigger study that seeks to enhance our understanding of how we can improve uptake of evidence in health policy development in Uganda. Previous work led to the development of a Middle Range Theory (MRT) outlining the main facilitating factors for translating evidence into policy [27]. MRTs are defined as “theories that lie between the minor but necessary working hypotheses (…) and the all-inclusive systematic efforts to develop a unified theory that will explain all the observed uniformities of social behavior, social organization, and social change” [28]. Our initial MRT detailing facilitating factors to the uptake of evidence was constructed around three elements that seemed particularly important in Uganda namely; the characteristics of the evidence, strengthened ministry of health (MoH) institutional capacity to lead the KT process and, existence of KT partnerships. Our MRT states the following:

“***High-quality and contextualized evidence will be taken up in policies so as to lead to evidence-informed policies in instances where the MoH leads the KT process and there are partnerships for KT in place.***

**Evidence** must be of high quality, contextualised, provide economically feasible recommendations, and produced in a timely manner by credible researchers. Use of local researchers is helpful but there is a need to separate the roles of researchers and policy makers.

KT requires **strengthened MoH institutional capacity to lead the KT process**. Institutionalized platforms for engagement between researchers and policymakers including civil society need to be in place, and mechanisms to coordinate evidence generation and synthesis need to be mainstreamed within the MoH. Policy makers need to be better at knowledge management and the policy-making process needs to be minimally bureaucratic.

**Partnerships for KT** need to be in place where all stakeholders are involved throughout the process right from evidence generation to application in order to improve trust and build interest. Communities need involvement in evidence generation and KT as well.

**These contribute to more ownership, adoption and better application of evidence***”*[27]*.*

This MRT was developed on the basis of literature review and validated through interviews with policy actors in Uganda without reference to a given research project and policy outcome. The extent to which the facilitating factors are valid in other settings needs to be tested on specific policy case studies.

### Selection of the case

The selection of the case we report in this study was guided by our initial MRT on KT [29]. The case on abolition of user fees seemed likely to predict contrasting results given the nature of the policy. Contandriopoulos et al. note that the characteristics of the policy have an impact on how stakeholders and policy makers consider evidence in the policy development process [30]. Whether the issue is polarizing that is, it is likely to cause fragmentation (high polarisation) among the actors involved given their positions on the issue under consideration, whether it is highly salient in that will attract a lot of attention and whether actors are familiar with the issue and as such it gains prominence on the agenda [30]. In addition, Moat et al. point out the need to understand how the context in which evidence is produced, the issues it addresses and issue-context resonance influence on the producers and users of evidence[17]. Regarding the context, the government policy making structures involved in policy making and the extent to which they are involving, the characteristics of political actors whether they stand to win or lose given the policy choices made and societal values, will impact on how evidence is viewed and taken up in policy development [17].

In the case of abolition of user fees, there were issue-context resonance factors that would impact the uptake of evidence. Polarisation tendency were eminent in that the actors were divided. For example, the World Bank (WB) was in for retention of user fees based on their concern to ensure loan sustainability, while the government was keen to address hindrances to realization of PEAP objectives**,** user fees for health care being one of them. It was a salient issue given that it is a social policy which directly concerns peoples’ welfare and this engenders a political currency reflected in popular support. Regarding context, Moat & Abelson in their analysis of the influence of institutions in the decision to abolish user fees in Uganda, concluded that “Big man” presidentialism and clientelism thwarted the role of formal institutions [31] indicating an exclusive policy making process. In this regard, the scientific rigor of the evidence may not be central in the decision making process.

### Background to the case study

The background to this study was described previously [8]. The policy process concerning user fees occurred within a given context, which impacted the decisions that were made. The period between the late 80s and early 90s was characterized by processes at both the global and national level that had a direct influence on the user fee policy. For example, the late 80s saw the global push of communities to take charge of their own health through the “self-help” drive, and subsequently the introduction of the Bamako Initiative schemes, which encouraged communities to contribute to the cost of health care [32,33]. At the local level, the country was just emerging from a civil war and the health care system needed rebuilding. The health sector reform programme developed at that time was unaffordable to the government, and potential donors expressed reservations due to the high cost required for its implementation. As one of the conditions for a loan to implement the programme, the WB proposed user fees, arguing that they would be key to ensuring sustainability. In addition, structural adjustment policies called for the subsidization of public service provisions by communities [34]. A second major event was decentralization. In order to bring services closer to the people, the country undertook decentralization reform in the early 90s, redefining roles and responsibilities between the central level and local governments (districts). The Local Government Act [35] allowed districts to charge fees for services they provided. Despite disagreeing with the introduction of user fees for health care at the level of parliament and the lack of an explicit policy on user fees, districts used the Local Government Act to institute their own fee systems. Implementation was patchy and poorly monitored, although some years later (in 1995), the Ministry of Health (MoH) developed guidelines [36]. The Highly Indebted Poor Countries (HIPC) initiative was the third main event. In the early 2000s, global actors pushed to reduce poverty in LICs. Uganda benefited from the HIPC initiative on the condition that the country invested debt relief funds into social services sectors, such as health. Uganda developed a Poverty Eradication Action Plan (PEAP) that prioritized investments in social services, including health. The country was keen to address all hindrances to the realization of the PEAP objectives, including user fees in public health facilities [37]. The early 2000s again saw the creation of a Sector Wide Approach (SWAp) in the health sector arguing for the alignment of all available resources to one agreed upon Health Sector Strategic Plan (HSSP) and more harmonization among donors [38]. As a result, more funding was realized to support the implementation of the HSSP [39].

The process leading to the abolition of user fees was characterized by protracted debates in cabinet, among technical officers in the MoH and among donors in their interactions with government without reaching a final decision. During the presidential campaigns in 2001, the President announced immediate removal of user fees for health care. Several reasons behind this decision have been hypothesized among which is, a sign of political commitment[40] as a strategy to secure votes given the proximity to elections [41]; or as a response to the findings of the Uganda poverty participatory assessment (UPPA) report [42].

The objective of this study was to explore the place of evidence in the design of the policy to abolish user fees in public health facilities in Uganda using a case study approach. We sought to assess the barriers and facilitating factors to the uptake of evidence in the policy development and, the extent to which the previously developed MRT explains the uptake of evidence from a policymaking perspective. In addition, we studied how the context and stakeholders involved shaped the uptake of evidence, building on earlier work in Uganda which assessed the role of actors in KT [43]. This study is part of a bigger study in which we are consolidating a MRT on KT in Uganda through testing of the initial MRT using multiple case design [44]. Eventually, the application of this MRT to concrete, selected health policy cases in an iterative manner will contribute to refining and enriching the previously developed MRT.

## Methods

### Over all methodological approach

#### Theory-driven inquiry

We adopted the theory-driven inquiry approach, which starts from the assumption that actors involved in any intervention (which should be considered to be broadly defined and encompassing any policy, strategy, or action plan) make a series of assumptions of how the intervention will work. Unearthing these assumptions is important because they help explain why actors make particular choices. Furthermore, these underlying assumptions can be compared to the existing body of evidence, the state of the art [45,46]. Eliciting this MRT (also called programme theory) not only helps us understand how the designers and implementers of an intervention think about it, but it can also be used as a hypothesis that can be tested on its explaining capacity. If done in a cyclical manner and, ever-refined insights are the result.

#### Case study approach

Given the need in theory-driven inquiry studies for in-depth investigation of the context, mechanisms of change, and actors, this study employed a case study approach [47]. Yin highlighted the importance of case study methodology in an investigation of real life situations in which boundaries between the phenomenon under investigation and context are not clear, and in which multiple sources of evidence are used [48]. Similarly, other researchers have used case study methodology to test theories in real life situations [49,50]. The present case study was performed between June 2012 and August 2013. The case is the policy processes related to abolition of user fees for health care in public facilities in Uganda, deliberations of which took place over a period of 8 years (1993 – 2001). Preliminary results of the case were presented to stakeholders prior to finalization.

#### Use of mixed methods

In a quest to improve the comprehensiveness and validity of the findings, the present study employed both qualitative and quantitative methods (QUAL + quant). Mixed methods are increasingly being applied to the investigation of complex issues in health systems research [51,52].

#### Identification of respondents and key documents for review

**Figure 1:**
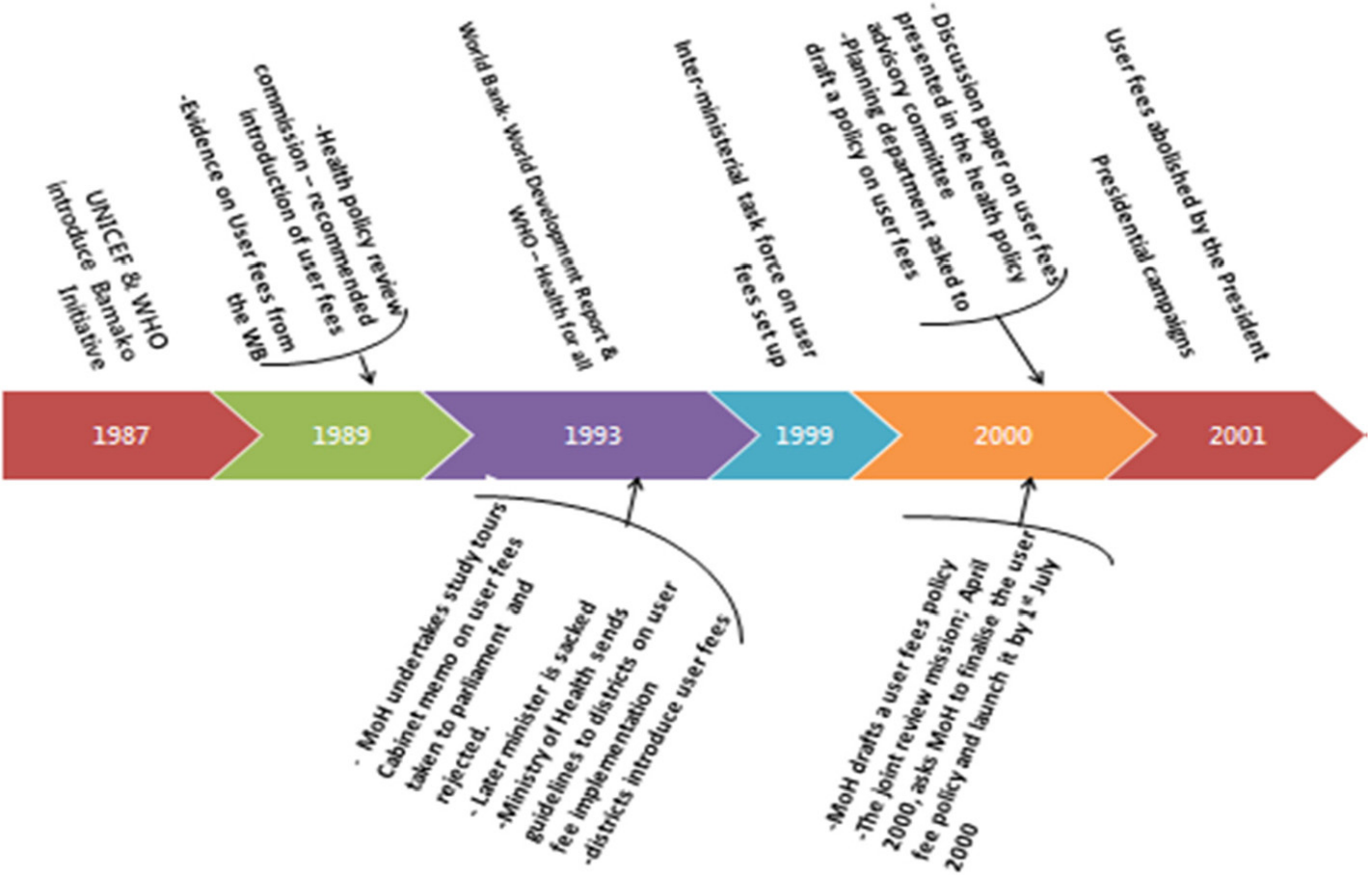
**Policy timeline.**

A policy timeline, which was drawn based on review of documents and, in consultation with focal persons on health financing (2 from the MoH, 1 from the WHO and 1 from the WB - who had been in post for over 15 years) guided the identification of milestones, selection of respondents and key documents to be reviewed (Figure 1).

### Selection of respondents

The institutions involved were identified and within these individuals central to the policy process were purposefully selected. The selection of respondents (Table 1) was further guided by the seniority of the individual and knowledge of the subject matter to be investigated [53]. Additional respondents were identified through snowballing until a level of descriptive saturation [54], some of whom had retired or changed employment. These respondents were categorized under the institutions they worked for during the policy change.

**Table 1:** **Key informants**

		**No.**	**Average years in post**
	Donors	3	10
Public sector	Ministry of Health	8	11
	Ministry of Finance	1	10
	District level		
	manager	4	9
	Service provider	4	7
	Parliamentarian	1	6
	Researcher in public university	1	8
Private sector	Civil society	4	9
	Journalist	1	8
	Service provider	4	6
	Researcher in a private institution	1	7
**Total**		**32**	

Two districts were selected based on proximity, presence of a regional referral hospital (Jinja district) or general hospital (Mpigi) to obtain perceptions from across the spectrum of health care delivery system. Within these districts, two hospitals and two lower level facilities (one public and one private not-for-profit in both cases) were purposively selected based on proximity and our desire to capture the different levels of the health care system. The selection of public and private not-for-profit health facilities was to help us understand the perception in a subsector where fees were eventually abolished (public) and a subsector where fees continue to be charged (PNFP). The medical superintendent or health center employee in-charge and one clinical staff member responsible for the outpatients department were purposively selected and interviewed at each health facility. At the district level, the district health officer and a member of the district health team in charge of supervising health facilities within the district were also purposively selected and interviewed.

### Selection of relevant documents

The timeline of key events guided the identification of relevant documents to be reviewed. We included a broad range of documents relevant to the case, to ascertain the processes involved the stakeholders, and their roles. The documents that were reviewed included the policy on user fees, discussion papers on health financing, the health financing strategy, budget framework papers, and research reports. Additional file 1 is the document review guideline and Additional file 2 provides details on the reviewed documents.

### Qualitative research methods

The qualitative part of the study was exploratory in nature and assessed whether evidence was available to guide policy decision-making, whether evidence was disseminated and discussed in relevant fora, and whether and why evidence had or had not been considered in policy development. This qualitative part of the study also assessed what respondents felt were the barriers or facilitating factors to the uptake of evidence. We used our previous MRT as the starting point [27]. Respondents’ perceptions were sought on the roles, level of support, and influence of the actors on the uptake of evidence.

### Data collection

Interviews were conducted with KIs using an in-depth interview guide consisting of open-ended questions. The interview guide was developed by the first author and was reviewed and refined by the research team prior to pretesting it with volunteer colleagues from different institutional perspectives (n = 5). KIs were contacted and invited by email or telephone to participate in the study. All identified respondents agreed to participate and were interviewed. All interviews were conducted by the first author in English and face-to-face (Additional file 3).

Relevant documents were reviewed to ascertain the type of evidence that was available, the extent to which evidence had been discussed in the different fora, and whether policy decisions aligned with the available evidence. Interviews with KI and review of documents were undertaken concurrently.

### Data analysis

Interviews, which lasted an average of 45 minutes, were recorded, transcribed verbatim, and entered into MS Word software for editing as the first step in the formal analysis. The transcribed interviews were enriched by additional notes made by the first author during the interviews. The first author read all of the transcribed interviews to identify emerging issues. We analyzed the data, both from the interviews and review of documents, using content analysis. We initially did a manifest content analysis and later a latent content analysis. In manifest analysis we elicited for (during the interviews) and looked for (in the data collected) elements and factors that were physically present and easily demonstrable and/or countable, in line with the study objectives, under different categories like the fact that evidence was used to inform budget discussions, politicians considered evidence, evidence informed cabinet discussions, evidence was used in lobbying for resources, and others. An examples of this is ……… *this evidence guided us in our discussions with the MoF, and the health sector budget increased significantly in an effort to cover the short fall from losses in user fee*s.... Furthermore we went a step further to do an interpretive reading of the symbolism underlying the physically demonstrable elements by doing a latent content analysis. We assessed participants’ feedback for evidence of facts like defending the evidence, advocacy for the government position, or advocacy for abolition of user fees – see Table 2. Stakeholders were identified as institutions. Drawing upon the work of Eden and Ackermann and Bryson [55,56], we classified the stakeholders using the influence/power – support/interest grid.

**Table 2:** **Example of content analysis process for the study**

	**Meaning unit**	**Category**	**Theme**
	Yes evidence has been used. We use evidence available at the time to compute how much was being collected and we compared this with the sector budget at the time. Operational research like the Uganda poverty participatory assessment undertaken by MoFPED, the Apuuli study on user fees that was commissioned by MoH were all used.	Evidence was used to inform budget discussions	Role of evidence in the policy process
	Although abolition of user fees was a political decision, at least there was some fury from the public that user fee had became politically unsustainable.	Politicians considered evidence	
	In the poverty participatory assessment study one of the biggest issues was the issue of user fees and access to services; so when this report came, it also caused worry among the politicians and we were asked to work out in financial terms, the cost of abolishing user fees. So we worked it out (......) we made recommendation to the minister and it was actually taken to cabinet and cabinet approved.	Evidence informed cabinet discussions	
	We used routine M & E data, a study undertaken by WHO and MoH (operational research). These sources of evidence showed increases in utilisation, drug stock-outs. We used this to lobby for increases in budget allocation. Districts also did some operational research whose results we used to lobby for more funding. The subsequent year, we had more money in the MTE.	Evidence was used in lobbying for resources	
	The truth of the matter is that this issue gets to be politicized because it was during campaigns that the president pronounced the abolition of user fees, but he actually used evidence from the district.	Politicians considered evidence	
	Regarding international evidence, this was mixed; some studies showed that user fees had some positive effects while others showed negative results. So there was lack of a firm position.	Contradictory findings	Characteristics of available evidence
	There were a number of discussion either on the benefits on chargers of user fees. But many of those studies were small based at the district level its after abolition that we were looking at levels of catastrophic expenditure; we were looking at the out of pocket expenditure in totality.	evidence was from small scale studies	
	Most of the studies on user fees in Uganda took place after its abolition.	Timeliness of the evidence	
	There was evidence but was that evidence properly synthesized? Was it properly shared by the different fora? Was it agreed that this was credible evidence for a decision to be made?	Questionable quality of available evidence	
	**Influence of stakeholders**		
	These were strong but their positions were mixed. The (X) were opposed to user fees. The (Z) from capitalist background were supportive of user fees. The (Y) had invested a lot in Bamako initiative so were supportive of the user fee policy. Actually in HPAC, there was no consensus.	Strong with mixed positions due to different reasons	Strong and divided
**Donors**	Donor (X) was in favor of abolition of user fees, they were even generating some evidence to show that user fees were a burden.	Strong and supportive of abolition of user fees	
	Some donors were for the abolition because the climate then was for poverty eradication and one of the things they needed to do was to help the poor to access health services. So they were saying that if now you have got the HPIC (debt relief), why are you complaining?	Strong and supportive of abolition of user fees	
	The big financial players like (X) were against provision of free services and they were bringing in all sorts of evidence some of which was good and some bad. Some donors led by (X) were just pushing it and they used whatever evidence they liked.	Strong, opposed and influential	
**Health workers**	They were opposed to abolition of user fees because they were beneficiaries.	were opposed because of the benefits	Strong and opposed
	The health workers were using the money initially to get themselves some extra income so how can they support that such an option is stopped?	Were opposed due to potential loss of benefits	
	Health workers were opposed to abolition of user fee because of the benefits. It was a steady reading available income, small as it may have been but it was there all the time. Now the challenge was, we were relying on them to implement the new free care policy.	Were against user fee abolition yet they were the ones to implement free care	

JNO, FS and BC reviewed and interpreted the findings. Converging issues were reviewed again by the research team and, when interpretation differed, consensus was achieved by revisiting the raw data and discussions. Identified regularities were compared with the previously developed MRT to identify convergent and other emerging issues, and the identified roles of stakeholders were compared with the previously developed roles of actors in KT in Uganda [43]. Similarities and contrasts between respondents’ perceptions were reviewed by the research team, and possible explanations for the contrasting views were discussed. When necessary, quotations that best represented emerging issues were edited slightly for flow while preserving the meaning of the text.

### Quantitative research methods

Quantitative methods were used to capture the multiple perspectives of the involved stakeholders and enable the identification of regularities and patterns [57].

### Data collection

The frequency, with which evidence was cited, including details on the type of evidence, was ascertained through the review of documents. In addition, KI were asked to rate the consistency between policy decisions and available evidence. Hanney et al. developed scales for rating the consistency between evidence and policy decisions in 2008 [24]; different parameters are rated on a scale of 1 to 4 (1 - considerable level of agreement, 2 - moderate level, 3 - limited level, 4 - no indication of consistency despite availability of evidence). Respondents were asked that: on a scale of 1 – 4, how would you rate the degree of consistence between the evidence that was available and the policy decision that was taken? They were further asked to give reasons behind their responses as a way of helping the research team assess the objectivity of a given rating. In applying the scales, the factors taken into consideration included: the extent to which the policy was consistent with evidence in terms of the definition of the policy problem, the definition of objectives, the description of the strategies and actions, and the extent to which the elements of the policy contradicted the available evidence.

A policy development framework including the steps agenda setting, analytical stage/policy formulation, decision making/selection of preferred options, and implementation [58] was used to organize the quantitative part of the case study.

### Data analysis

Quantitative data were analysed using Excel spreadsheets.

Findings from the review of document and KI interviews were integrated throughout the analysis. In addition, qualitative and quantitative data were eventually triangulated.

Informed consent was obtained from all respondents prior to the interviews. Study participants were informed about the purpose of the study and the scope of issues in the in-depth interview guide. Confidentiality was ensured in data management and only aggregate information without subject identifiers is reported. All data were secured in a safe location accessible only to the study team. Ethical approval was obtained from the Institutional Review Board of the Institute of Tropical Medicine, Antwerp (Belgium; IRB number IRB/AC/ac/197) and the Uganda National Council for Science and Technology (number SS 2920).

## Results

### Qualitative results

Evidence informed decision making in different ways. The MoH institutional capacity to lead the KT process, the partnerships for KT, the political context and the overall government discourse impacted the uptake of evidence.

### Role of evidence

The nature of the available evidence reported by the different respondents as having been available is shown in Table 3, although majority of respondents mentioned community complaints as evidence that was available.

**Table 3:** **Type of evidence that was available as reported by the respondents**

		**Community complaints**	**Operational research**	**International evidence**	**Routine M & E**	**Surveys**	**Experience from pilots**
	Donor	2	2	2	1	1	1
Public sector	Ministry of Health	5	5	3	4	1	1
	Ministry of Finance	1	1		1	1	
	District level						
	manager	3	1	1	1		
	Service provider	3	1	1	1		
	Parliamentarian	1	1	1			
	Researcher in public university	1	1	1		1	1
Private sector	Civil society	3	2			2	
	Journalist	1	1				
	Service provider	2	1		1		
	Researcher in a private institution	1	1	1		1	
	**Total**	**23**	**17**	**10**	**9**	**7**	**3**

From the late 80s to the early 90s, international evidence was mainly available from the WB, United Nations Childrens’ Fund (UNICEF), and the World Health Organization (WHO). In the mid 1990s, the MoH turned to other countries to learn from their experiences in health financing. Evidence was also available from surveys, more specifically the UPPA study that aimed to inform development of the PEAP.

Evidence informed decision making in several ways. Instrumental use of evidence was reported when the MoH used evidence in their dialogue with the MoF to determine the budget allocation to the health sector, as stated by a MoH respondent, “*We used routine monitoring data to develop scenarios of how much was being collected from user fees. This evidence guided us in our discussions with the MoF, and the health sector budget increased significantly in an effort to cover the short fall from losses in user fees*”.

Some respondents argued that, although the abolition of user fees coincided with political campaigns, the president actually used evidence in the form of community complaints: *“the truth of the matter is that this issue gets to be politicized because it was during campaigns that the president pronounced the abolition of user fees, but he actually used evidence from the districts. People cried and said ‘user fees for health is not for the poor’. So I still say that there was evidence based on reporting from the field through political rallies.” (*MoH respondent)

Symbolic use of evidence was reported when the president used evidence to make a politically attractive decision given the impact user fees for health on peoples’ welfare, as a MoF respondent stated, “*Evidence, which was mainly from UPPA surveys, could have been an important input but I think there was clearly a very large input that came out of the fact that it was a policy likely to win a lot of population support; where many people would be affected and, for the politicians, it made sense*”.

Conceptual use of evidence was also reported where by evidence informed discussions in cabinet. The findings from the UPPA raised a concern among politicians who then requested for more evidence, on the financial implications of abolishing user fees. Results of the study were tabled and discussed in the cabinet.

A review of documents revealed that the abolition of user fees was a policy likely to win popular support, and in the briefing paper on user fees, such a policy was mentioned to be a consideration at the launch of the new 5-year sector programme.

Nonetheless, some respondents (n = 5) argued that evidence was not used in an objective manner, as researcher stated that **“***I would say, yes, evidence was used but not in a manner that was very helpful. First of all, the evidence that was generated and upon which user fees were abolished, in my view, is a little bit questionable because it did not cover the extensive opportunities that existed with user fees. Not everything was considered and put into context.”* (Researcher respondent)

From our interviews, the ills of user fees for health care were clearly known given the available evidence from surveys like the UPPA and routine monitoring data on health service utilisation, but the planned course of action to address the problem differed between technical (MoH) and political wings. Though the MoH had a plan to phase out user fees for specific services, the political wing opted for free health services. A MoH respondent stated that “*the expenditure on health had a huge component of out of pocket expenditure and we were going to deal with this problem. We put down that evidence and we proposed how it should be phased out, but before that took place, a political pronouncement to abolish user fees was made*”.

### Factors that impacted the uptake of evidence

#### Available of evidence

Evidence was available although, concerns regarding the quality, comprehensiveness, objectivity, and timeliness were raised, which impacted its uptake into policy. The timeliness of the evidence varied; some respondents reported that evidence was available while others reported that evidence was only generated to justify a decision that was already made. According to a donor representative, *“Most of the research studies on user fees in Uganda took place after its abolition.”* A service provider in a public facility stated that “*some groups of people came to ask questions about user fees but this was well after the government had made the pronouncement to abolish it*”.

A review of documents also revealed the varied timeliness of evidence. Some studies took place before the abolition of user fees while others were conducted after the policy decision.

Evidence existed for and against user fees, which to some extent was interpreted as contradictions. One MoH respondent stated that “*some studies showed that user fees had some positive effects while others showed negative results. So there was lack of a firm position.*” A donor respondent added, “*I don’t think we had very convincing evidence at that time. There was evidence for and against user fees and the quality of the evidence was also questionable*”.

The objectivity of some evidence was a concern as several of the available studies had been undertaken by donors, and these were judged as misleading. According to a MoH respondent, *“The MoH had been misled by the supply side evidence generated by [X] of the WB, who showed that when you abolish user fees, increases in access would not be significant”*.

The comprehensiveness of the local evidence was also in question, as a journalist stated, “W*e did have some complaints from the community that people were staying away because of user fees but that evidence was not enough to guide us on the way forward. Although these were also echoed in the UPPA study, we should have done a thorough study to assess how the removal of the user fees would help because some of these problems have persisted even when the services are free. Community complaints should have been a starting point for a study and not a basis for decision making.*” In addition, a MoH respondent remarked that “*although some evidence was available, it was not enough and also not credible*”.

Technocrats made an effort to disseminate evidence to politicians, as pointed out by a MoH respondent, “*We were reading these reports, synthesizing them and bringing evidence as digestible bits to politicians. They would take it or leave it depending on what positions they wanted to take.*” Some of the recommendations made by technocrats were actually tabled in cabinet, as reported by a MoH respondent: “*we wrote a policy brief for cabinet showing them how much the government had to invest in order to reach the level made by user fees. We made recommendations to the Minister of Health and these were taken into consideration*”.

### MoH institutional capacity to lead the KT process

Weaknesses in the capacity of the MoH to lead in evidence generation, synthesis, and application were reported. The generation and dissemination of evidence was mainly donor driven, with the MoH playing a recipient role, as pointed out by a MoH respondent: “*big players like the WB were just pushing their agenda, bringing in all sorts of evidence, some of which was good and some was bad, they used whatever evidence they liked. It is not that people were sitting down and deliberating.*” However, respondents reported that, in one of the local studies, a team headed by a researcher from a public university was put in place, with the MoH serving as the secretariat, to collect data on the various aspects of user fees.

A review of documents confirmed that the inter-ministerial task force on user fees was headed by a researcher and the MoH was the secretariat. Later, a health financing task force, involving MoH official, donors and CSO representatives was put in place to develop policy options on improving health sector financing and was chaired by the MoH.

Although leadership at the MoF was strong, the MoH suffered a high turnover at the senior officer level, which weakened its leadership capacity. The leadership in the MoF was reportedly able to synthesize the evidence, as a MoH respondent stated, “*Strong leadership at the MoF was a key issue - the leadership was listening to evidence.*” In the MoH, the leadership went through times of strong and weak leadership over the policy timeline for user fees; a MoH respondent stated that “*at one point we had a strong leader and at another time we had a consortium of leaders with other interests, so they were not looking out to see what needs strengthening.*” In times of strong leadership, leaders in the MoH were reported to be visionary: “*there was a culture of questioning whether we were doing things right all the time, we would look at evidence from research studies, surveys and routine monitoring.*” (MoH respondent)

### Partnerships for KT

KT partnerships are key to improving the uptake of evidence, and these were reported to be in place although several weaknesses were reported regarding their membership, duration, and scope of work. A one-off task force was put in place to coordinate the generation, synthesis, and dissemination of evidence to decision makers, including the cabinet, although it mainly consisted of senior MoH staff, MoF staff, researchers, and the WHO. Donor involvement was only reported in the development of guidelines; the MoH respondent stated that “*there was a lot of work to be done to produce guidelines on exemptions for those who could not pay and we requested support from UNICEF.*” Community involvement on the other hand was limited to being respondents in surveys.

In addition, a review of documents revealed that throughout the evidence generation and policy discussions, only two task forces were put in place. These task forces were short-lived and only performed specific tasks. The inter-ministerial task force on user fees was tasked with reviewing and synthesizing available data and providing policy options. The working group for health financing convened after the abolition of user fees to provide policy options on improving health sector financing.

Decision making was reported as non-consultative; one MoH respondent stated that “*there was no consultative process in the decision to abolish user fees*”.

### Political context

The year in which user fees were abolished, 2001, was marked by presidential elections, and the different candidates were moving around the country canvassing for votes. The issue of improving service delivery and access to health services, specifically the abolition of user fees for health care in public facilities, was in the manifesto of both the ruling party and the leading opposition party. The ills of user fees had been highlighted in the UPPA study whose findings were disseminated widely by the media and CSOs and, even discussed in political circles. A majority of respondents reported that this political window could have provided an opportunity to abolish user fees. According to a MoH respondent, the *“Abolition of user fees was in the election manifesto of the ruling party and this showed that they were responding to population concerns.”* Second, the community presented the ills of user fees for health care to the president while on a campaign trail, as noted by a CSO respondent: “*complaints about user fees were raised to the president during campaigns where he had to offer an incentive to have more votes.”* A private not-for-profit service provider further added that “*there were community cries about the ills of user fees and indeed the scrapping of user fees was announced at a political rally*”.

The opposition candidate was promising to scrap user fees for health care once elected to office and the pronouncement by the president could have been a tactic to pre-empt the opposition manifesto. A MoH respondent stated, *“I suspect the pronouncement to abolish user fees was through political pressure and competition because the main opposition party was saying ‘we will abolish user fees for health services immediately’ so the captain could not say ‘I will wait for the plan’, they could lose votes”.*

Another MoH respondent argued that evidence was taken seriously because of the political context: *“evidence by itself would not have constituted sufficient force to enable the government to make the decision, but because they were under political pressure, evidence gave them reason to make the decision to abolish user fees”.*

The political process impacted the KT process in that it did not allow enough time for the diffusion of evidence, as stated by a MoH respondent, “*Political experience overtook this process and the decision to abolish user fees was not a health sector decision; it was a pronouncement from the head of state. We had not gone to him and given him evidence directly, but communities had complained to him.*” A parliamentarian added that the “A*bolition of user fees was a political decision, it was not based on systematic evidence but there was some fury from the public that user fees had become politically unsustainable*”.

### Overall government discourse

There was a move at the global and national level to eradicate poverty and the country wanted to address all issues that would hinder realization of the objectives of the PEAP. A MoH respondent stated, *“The Uganda poverty participatory assessment (UPPA) showed that user fees were a hindrance to accessing care and also to poverty alleviation efforts. This was at a time when the government was keen to reduce poverty and we were writing poverty reduction support credit papers”.*

### Barriers to the uptake of evidence

Inadequate funding to implement evidence was cited as a barrier by a MoF respondent: “*one of the barriers was the limited fiscal space for giving free public goods in all sectors.*” In addition, although services were free, there were gaps in service delivery, as a MoH respondent stated, “W*e saw a tapering off of patient utilization when the community realized that a number of health facilities did not have sufficient supplies of essential medicines so it was useless going for free things when they are actually not there.*” Poor planning for the policy change was reported by a donor respondent: “*We didn’t put in place proper system structures that when user fees are abolished, something replaces them to ensure continuity of services*”.

### Roles, level of influence and support of actors in the uptake of evidence

Stakeholders played different roles in the uptake of evidence (Table 4). The MoH played multiple roles, including generating of evidence by working with researchers, disseminating evidence, and implementing policy decisions. In addition, the MoH played advocacy roles. As a MoH respondent stated, “*The ministry’s role was advocacy, defending the position of the MoH, and persuading other people to join us”*.

**Table 4:** **Roles of stakeholders in the uptake of evidence**

	**Stakeholder**	**Roles**
**Public sector**	Ministry of Finance	Providing funds, generating evidence, engaging in policy development
	Ministry of Health	Generating evidence, disseminating evidence, advocacy, implementing the policy
	Managers at the district level	Implementing the policy
	Service providers	Implementing the policy
	Researchers in public universities	Generating evidence
	Donors	Generating evidence, disseminating evidence, providing funding , advocacy
**Private sector**	CSOs	Generating evidence, disseminating evidence, advocacy
	Community	Advocating for user fee abolition, beneficiaries of the policy change
	Researchers in private research institutions	Generating evidence
	Media	Disseminating evidence

The roles of donors were reported as providing funding and generating evidence. Some respondents reported that influential donors dominated the process of evidence generation and decision making about user fees. The CSO respondent stated, *“The WB had all sorts of publications on user fees and was making the argument that user fees need to be included in social service delivery areas like health and education.*” Respondents reported that the debate on user fees started after a WB publication showing that the introduction of user fees would not affect the utilization of health services. User fees were introduced as a demonstration project of the WB. A MoH respondent stated, “*We had started decentralization and in each of the thirteen districts we were going to introduce user fees for health care as a pilot and demonstration project of the WB*”.

CSOs played a role in generating evidence; a CSO respondent stated that “*CSOs generated evidence which showed that, when user fees are charged for health care, the rural and urban poor are disadvantaged.”* Respondents also highlighted the role of CSOs in disseminating evidence; a parliamentarian respondent stated that “*CSOs produce policy briefs and distribute them. They find people with influence or who are close to policy makers and put them on their list and every time they find new ideas, new findings, new research, they just disseminate it through their network, and I usually pick a lot of those.*” The advocacy role played by CSOs was highlighted by a researcher who stated, “*I think their advocacy role helped create sufficient pressure for the government to abolish user fees*”.

The role of the media in disseminating evidence was highlighted by a CSO respondent: “*we used to have newspaper articles highlighting the barriers to access due to user fees.*” However, a donor respondent decried the under-utilized role of the media stating, “*I don’t think we use the media well enough. Sometimes journalists sit outside the offices of some decision makers and fail to see them and that’s why they end up writing whatever stories they have. Once a journalist mentioned to me that he went and sat outside [X’s] office and was not given an opportunity, and yet with the politicians, they do it very well; they will always want to send their issues across.*” A MoH respondent cautioned about the possible negative role of the media, stating that “*if the media do not base their publications on evidence, they can be a very detrimental ally*”.

**Figure 2:**
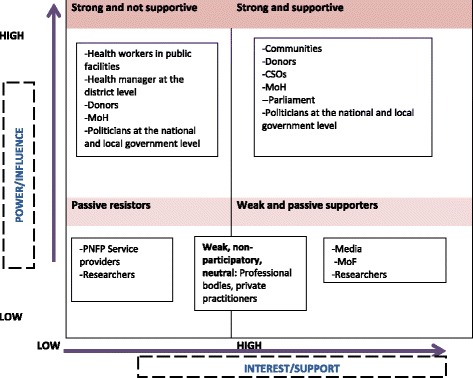
**Positions of stakeholders involved in the policymaking process for the abolition of user fees.**

The level of support and influence by the different stakeholders on the decision to abolish user fees in light of available evidence varied as shown in Figure 2 but, for different reasons. The strong stakeholders were characterized by significant funding, the power of the vote, and being key decision makers. Several respondents were divided; for example, some donors were reported as having been strong and influential supporters of abolishing user fees due to their strong considerations for the poor. Evidence had showed the poor could not access services due to cost [42]. Yet others were strong and not supportive due their ideological background; for example, the WB believed that health care should be part of the market and indeed had generated evidence showing user fees would not affect utilization of services adversely [59], whereas others were opposed to this because of prior institutional positions on user fees, such as UNICEF and the WHO because they had invested a lot in the Bamako initiative which showed some promising results regarding improved availability of medicines and increase in utilization of health services [60]. A MoH respondent stated that “*donors were strong but their positions were mixed. The [X] from pro poor systems were opposed to user fees while the [X] from a capitalist background were supportive of user fees. WHO and UNICEF had invested a lot in the Bamako initiative so were opposed to user fee abolition. Actually, in the health policy advisory committee, there was no consensus”*. (MoH respondent)

The MoH was also divided in that some officers were strong and supportive of abolishing user fees but others were strong and opposed. Although the officers who were strong and opposed were equally concerned with the evidence which showed a high out of pocket expenditure on health [39], their course of action was reported to be different, as one researcher stated, *“The MoH was working on a cabinet memo to reorganize user fees but after 2 months, when presidential campaigns were ongoing, I heard a speech from the president saying ‘I have never supported user fees,’ he even said he will not support user fees, so the minister who had championed the whole user fee issue had to write memos to stop it”.*

Communities were strong supporters of the abolition of user fees, and influential given the political window as reported by a MoH respondent: “*communities put the issue of the ills of user fees for health care on the agenda, they were strong in that they are the electorate and the president wanted votes. They kept complaining. They were supportive of the abolition of user fees and they were strong.*” A researcher added, “*If the voters were that important then, yes, they had influence,*” and a service provider remarked that “*the community was in full support of user fee abolition because it was to their advantage.*”

However, a civil society respondent highlighted a contrary view that the rich in the society were not supportive of abolishing fees for health, stating that “*people who had the willingness and ability to pay were feeling that ‘if you take away user fees, that means that I may not get the same quality, yet am willing to pay, I have money’.*” (CSO respondent)

CSOs strongly supported the abolition of user fees, although the reasons behind their support varied. Some had concern for the poor, as a MoH respondent stated, “*The CSOs supported the abolition of user fees. We had [X] on some of those working groups and they were really pushing for the abolition of user fees for health care, saying that evidence showed that poverty was biting.*” This was in reference to the evidence from the UPPA and CSO’s own research. Some CSOs were supportive because they believed there was enough money to fund health services without communities paying, as one donor respondent stated, “*The CSOs were against the user fees because they believed that there is a lot of money invested in the health sector and there was no reason for people to pay in order to access health services.*”

Respondents reported that politicians at the national level had divided positions, stating that some supported the abolition of user fees because of potential political gains, whereas others opposed it because they were concerned about service delivery given the evidence of increased availability of medicines as a result of charging a small fee for services [61,62]. A donor respondent stated that “*politicians had a divided opinion, some supported user fee abolition for the purpose of popularity, especially votes. Some were for user fees because they believed that user fees could improve service delivery by offering a small allowance to health workers, which boosted their morale*”. The improved availability of drugs and incentives for health workers was also shown in some pilots supported by WHO [60].

Politicians at the local government/district level were also divided; some were strong and opposed to the abolition of user fees due to the potential loss in revenue and the preference to generate local revenue that could be managed at the district level rather than receiving grant from the central government s. A CSO respondent noted that “Y*es, they were opposing the abolition of user fees just as they are still opposing the abolition of graduated tax, yet they are getting almost 10 times the money they used to collect from graduated tax! They want something they can independently manage*”.

Managers of health services at the district level and service providers in public health facilities were reported as strongly opposing the abolition of user fees and were influential because the implementation of the policy heavily depended on them; they were opposed because of the potential loss in revenue.

Although researchers were reported as being less influential, they were also divided, with some supporting and some opposing the abolition of user fees. The division was partly reported to be due to the kind of evidence they were generating. A researcher stated that “*researchers were divided, some were for abolishing user fees, some were not, and there was evidence on both sides.*”

### Quantitative results

Various types of evidence were cited in strategic documents and discussion papers as shown in Table 5. Local evidence as opposed to international evidence; operational research and evidence from surveys were cited most.

**Table 5:** **Type of evidence cited in the reviewed documents**

**Nature of evidence**	**Local evidence**	**International**
M&E	5	
Operational research	7	
Regular surveys	9	
Costing studies by the World Bank		2
Local costing studies	2	
Evidence on effects of user fees on utilization of health services and drug availability provided by WHO & UNICEF		4
Evidence published in Journals	1	
**Total**	**24**	**6**

Respondents ranked the degree of consistency between policy decisions and available evidence as weak at all stages of policy development (Table 6). Better consistency was reported at the agenda setting stage than at other stages. The consistency between available evidence and policy decisions was rated the weakest and as having no influence, mostly at the implementation stage.

**Table 6:** **Rating of the consistency between available evidence and decisions**

			**Public sector**			**Private sector**			
		**Donors (n = 3)**	**MoH (n = 6)**	**MoF (n = 1)**	**Service providers and managers at district level (n = 6)**	**CSOs (n = 4)**	**Researchers (n = 2)**	**Service provider PNFP (n = 3)**	**Total**
**Agenda setting**	Strong (1)		3	1		1	1		6
	Moderate (2)		1						1
	Weak (3)	3	1			3			7
	No influence (4)		1				1		2
**Analytical stage**	Strong (1)		2						2
	Moderate (2)		1			1	1		3
	Weak (3)	3	2			2	1		8
	No influence (4)		1			1			2
**Decision making**	Strong (1)		3						3
	Moderate (2)			1					1
	Weak (3)	3	1			3	2		9
	No influence (4)		2			1			3
**Implementation**	Strong (1)		1						1
	Moderate (2)		1	1	3			1	6
	Weak (3)	3	2		1	2	1	2	11
	No influence (4)		2		2	2	1		7

Service providers could only rank at the policy implementation stage.

MoH: two were not able to rank and were excluded from this analysis.

Service providers: three were not able to rank and were excluded from this analysis.

## Discussion

The present study highlights the ongoing debate on what constitutes evidence and gives good examples of how evidence may be considered, used, or even misused in the policy development process. Our findings show that evidence informed decision making in different ways. The MoH institutional capacity to lead the KT process, the partnerships for KT, the political context and the overall government discourse impacted the uptake of evidence in the abolition of user fees for health care in public health facilities in Uganda.

The process of abolishing user fees was informed by evidence generated from formal research but also from community complaints. Though peer-reviewed research has been considered the most appropriate evidence in the past [63], it is now becoming clear that there are many forms of evidence that inform policy and decision making [12]. What is taken as evidence may vary from case to case due to the stakeholders involved and how much interest and influence they wield. In our study, we have noted that different actors are influenced by different types of evidence, the WB relied mostly on formal research as the basis to push for the introduction of user fees, technocrats in the MoH relied mostly on formal research to push for reorganization of user fees while politicians relied mostly on community complaints to abolish user fees. Not only is the definition of evidence debatable, but also how much evidence is enough and whether it is seen as comprehensive [64]. Though some respondents in our study were comfortable using community complaints as a basis for decision making, others argued that they should only have served as a basis for more formal research.

In our study, evidence informed policy and decision making in different ways. Some respondents stated that evidence was used to support a decision that was already made, implying ‘symbolic use’, whereas others reported that available evidence was presented in cabinet to guide discussions, implying ‘conceptual use’. Yet others reported that the analysis from losses in revenue if user fees were abolished directly guided budget allocations to the health sector, implying ‘instrumental use’.

The characteristics of the available evidence may have impacted how evidence was used in several ways. Concerns were raised regarding the quality of the available evidence, the contradictory conclusions from the research studies, and the limited dissemination of evidence, which are known barriers to the uptake of evidence [24,65]. The fact that most research studies were undertaken by the WB, whose ideology was in line with the recommendations drawn from their studies, raised questions regarding the objectivity of such evidence, and some respondents mentioned that this evidence was judged as somewhat misleading. The dissemination of available evidence suffered shortcomings due to a lack of mainstream mechanisms from the MoH to coordinate the process. Technocrats in the MoH focused on disseminating evidence to the cabinet through policy briefs and cabinet memos, and donors and CSOs were also disseminating evidence generated from their studies, whereas communities were complaining directly to politicians. The timeliness of the evidence also varied; some respondents reported that there was evidence regarding the ills of user fees, and others reported that evidence was generated after the decision to abolish user fees was made. The varied timeliness and uncoordinated dissemination could explain the several forms of evidence use noted in this study in that, the uptake of evidence is influenced, to some extent, by the nature and timeliness of the evidence and how and to whom evidence is disseminated. For example, politicians received community complaints during campaigns and they used it symbolically because it favored their interest, whereas technocrats received evidence at the time of the budget process and they used it instrumentally in determining sector allocations.

The MoH’s institutional leadership of the KT processes was weak; the process of evidence generation and dissemination was mainly donor driven with the MoH playing primarily a recipient role. We noted a lack of coordinated efforts to synthesize and disseminate evidence, which could have served to resolve the seeming contradictions in the available evidence. This finding may explain the different course of action between the technical (MoH) and political (cabinet) players in addressing the high out of pocket expenditures on health; the technocrats at the central level MoH preferred a re-organization of user fees, and politicians preferred the provision of free health services. In addition, the high turnover of senior positions in the MoH further resulted in weaknesses in leadership due to lost institutional memory and having leaders at certain times who had minimal interest in the use of evidence. The high turnover of senior officers has been highlighted as one of the limitations of the enlightenment model, which infers a notion of gradual sedimentation of ideas over time and the subsequent uptake of evidence in policy [21,66]. Policymakers may not stay in positions long enough to allow for the gradual sedimentation of ideas. The significant dependence on donor aid to finance health services [67] could also have weakened the negotiating power of the MoH in that, even when they had different views on possible courses of action given the available evidence, they could not go against the donor influence. The introduction of user fees was a prerequisite to obtaining a loan from the WB. Moat & Abelson further highlight the challenge of what they characterized as policy a legacy in their analysis of the influence of institutions in the decision to abolish user fees in Uganda. They argue that in a quest to access a WB loan for the health sector reform programme, the government conceded to the demands of the WB allowing them significant authority over domestic policy [31]. Donor dependency is a documented challenge, as in the case of Cameroon, where donor dominance undermined the country’s efforts to coordinate research [68], and the case of Ghana, where changing HIV treatment guidelines was largely influenced by the conditions of donor financing [69]. Systematic platforms for engagement between researchers and policy makers have been shown to be beneficial in KT [70,71] but in this study these were informal and weak.

The consistency between available evidence and policy decisions was highest at the agenda setting stage, decreasing thereafter, implying that the political process may not have allowed enough time for the diffusion of evidence. The lack of a consultative process in decision making was found in this study. Although a challenge that cannot be overlooked is how consultative platforms within which dialogue may occur can work alongside time-bound political processes. Moat & Abelson in their analysis of the influence of institutions in the decision to abolish user fees in Uganda, highlighted the weaknesses of government structures to impact on the decision making process to abolish user fees concluding that “Big man” presidentialism and clientelism thwarted the role of formal institutions[31]. This may explain why the symbolic use of evidence features significantly.

There were some KT partnerships in place, though these were short lived with time-bound assignments. Effective partnerships for KT require mutual trust [70,72], but respondents in the present study noted that some groups expressed a preference for certain pieces of evidence because they supported particular positions, ‘misleading’ decision makers. Such mistrust leads to evidence not being used or only minimally used because of skepticism. No matter how good the evidence is, if there is mistrust it will be seen in the same light, and that presents a missed opportunity for KT.

Although funding was made available as part of debt relief, it was inadequate and hampered the uptake of evidence. A tapering off of service utilization due to a lack of essential medicines has been reported in other studies as well [8]. Other researchers also documented a lack of funding and required inputs as a hindrance to the uptake of evidence [64].

The different actors played different roles and had varying levels of support and influence. For example, the MoH played an advocacy role previously reported by CSOs and politicians in an earlier study on the roles of actors in KT in Uganda [43]. This change can potentially be explained by the MoH seeking public recognition, as the abolition of user fees is a policy that appeals to a larger section of the population due its welfare effects. On the other hand, the abolition of user fees for health care was a politically appealing policy, and the technocrats in the MoH may have had political inclinations. Donors played several roles in the dissemination of evidence and advocacy. In an earlier study on the roles of actors in KT in Uganda; donors’ roles were reported as providing funding for undertaking research and implementation of research recommendations [43].The finding in this case study may be explained by the nature of the donor agencies involved; common to all of them was that they had institutional positions regarding the policy under discussion and, thus, the tendency to push agency positions. The perceived tendency to rally behind an institutional position can impact the KT process, a concern that was raised by respondents in this study when they questioned the credibility and objectivity of evidence generated by some actors. The question of how evidence can be used objectively amidst institutional agendas and donor conditions in aid-dependent countries remains unanswered.

Regarding stakeholders’ level of support and influence, almost all stakeholders were divided, but for varying reasons. Divided views were found among actors on whom funding decisions and the successful implementation of evidence heavily depended. Consensus is however important for the successful implementation of evidence; for example, prioritized resource allocation to implement evidence can only be realized with an agreed upon course of action [73]. In our study, some respondents noted a suboptimal implementation of the policy to abolish user fees, which has also been documented in other studies [8].

The political context within which the abolition of user fees occurred also played a role in the uptake of evidence. The timing of presidential elections provided an opportunity. This case is a clear example of Kingdon’s problem of political and policy streams meeting up to form a policy window [74]. In the problem stream, there was agitation to try to get policy makers to focus on the problem of user fees. This had been raised from UPPA surveys whose results had been disseminated extensively. Similar agitation also came from the community as complaints. Such agitation was presented to politicians, including those campaigning for the presidency at the time. In the political stream, the issue of user fees for health care was in the election manifesto of the two presidential candidates. Yet the policy stream did have several proposals that were ongoing and were in fact advocated, like the World Bank’s proposal to make health care part of the market where one pays to get what they want. Presidential elections provided an opportunity for the three streams to meet and a window presented itself for a policy decision on user fee removal. As one respondent commented, *“…if there was no pressure of any kind, evidence by itself would not have constituted sufficient force to enable the government to make the decision.”* The political window is a documented facilitating factor to the uptake of evidence [19,74]. In addition, user fees for health care were a hindrance to achieving the objectives of the PEAP, which was also reported to have influenced the policy decision. Maja de Vibe et al. noted that ideas and concepts are more likely to be chosen if they are in line with the dominant policy discourse and serve to confirm agreed upon approaches [74].

### Comparison with our previously developed MRT

There were similarities as well as differences regarding the factors that impacted uptake of evidence looking at our initial MRT and the factors identified in this study. Regarding the characteristics of the available evidence, although the availability of evidence was crucial, its timeliness, scientific rigor, credibility of researchers and separation of roles between researchers and policy makers, as identified in our initial MRT, did not feature prominently in this case study. Regarding the MoH institutional capacity to lead the KT process, although platforms for engagement were in place, they were short lived, weak and of limited involvement. Mainstreamed mechanisms (within the MoH) to coordinate evidence generation, synthesis and dissemination were absent. Regarding partnerships for KT, although these were in place, they were informal, limited in scope and membership. These weaknesses with standing, we note that there multiple uses of evidence in the decision making process.

The multiple uses of evidence and lack of emphasis on the scientific rigor of the available evidence can be explained by the nature of the case under study. Even when evidence is available, the success of efforts to effectively disseminate, legitimize and consider evidence objectively in policy development will be impacted on by the characteristics of the issue under consideration [30]. This could be explain by what Contandriopouls et al. categorize as polarizing characteristics of a policy issue where In cases of low issue polarisation, potential users of evidence share similar opinions and preferences, they all see the issue as a problem, discussions are more likely to be technically focuses and instrumental use of evidence realized [30]. On the other hand, issues of high issue polarisation have a tendency to cause fragmentation given the position of actors involved; discussions are likely to be entangled in political debates and unbalanced power plays and in such instance, symbolic use of evidence in more likely [30].

In the case of abolition of user fees for health care, the high issue polarisation was noted. The WB was in support of user fees based on their concern to ensure loan sustainability**;** the government was keen to address hindrances to realization of PEAP objectives user fees for health care being one of them. CSOs were in support of abolition of user fees to improve access to health services for the poor, managers and service providers at decentralized levels were opposed because they stood to lose a source of revenue and politicians at the central and decentralized level stood to lose votes. Contandriopouls et al. further state that if an issue is highly salient in that it will attract a lot of attention and; whether actors are familiar with the issue thus it gains prominence on the agenda [30,74] will impact the uptake of evidence. The case of user fee was highly salient given that majority of the population were categorized as poor [75] and stood to benefit from the abolition of user fees, indeed community complaints featured strongly. This high salience nature of the issue was also evidenced in the media interest as noted by a respondent who stated that “*we used to have newspaper articles highlighting the barriers to access due to user fees”.* These may explain the multiple uses of evidence, lack of consensus on the available evidence and preferred course of action and; the lack of emphasis the scientific rigor of the evidence.

### Refined MRT

We refined the initial MRT as follows.

“Evidence will be taken up in policies so as to lead to evidence informed policies in instances where the MoH leads the KT process, there are partnerships for KT in place and, the nature of the policy issue under consideration, the overall government agenda and; the political situation can be expected to play a role”.

Evidence must be available and disseminated to stakeholders and;

KT requires strengthened MoH institutional capacity to lead the KT processes and platforms for engagement between researchers and policy makers including civil society need to be in place.

Partnerships for KT need to be in place. There is need to devise mechanisms for stakeholder engagement and resolving conflict of interest. Communities need involvement in evidence generation and KT as well.

A favorable political context in which a political window provides an opportunity for KT and;

Evidence aligning with the overall government policy discourse, enhance uptake.

These contribute to more ownership, adoption and better application of evidence.

### Strengths and weaknesses of the study

The strength of our study is the diversity of respondents, which provides a rich set of information to study the case. In addition, we have used multiple sources of data (interviews and document review) and mixed methods which have increased the validity and reliability of our findings. The multidisciplinary nature of our study team also strengthens the study given the ability to analyze and interpret data from multiple perspectives.

Among the weaknesses of our study is recall bias, as the KT processes being studied occurred took place some time ago. However, we think that this is not so much of a limitation given the consistency in responses. The direct impact of the abolition of user fees on peoples’ welfare, which makes it politically attractive, may have affected the nature of responses in cases where some respondents may have had political inclinations, but this was not assessed in our study. We have used scales developed by Hanney et al. in rating the degree of consistence between the available evidence and policy decisions taken. Although this work was been published [24], the rating scales have not been validated in low income settings. Although different types of evidence were available, community complaints, which are not categorized as systematic evidence, seem to have influence the decision of political actors. We did not study the influence of the different types of evidence on decision making because we believed that the different types are interrelated and inform each other passively. For example, expert opinion by definition is based on evidence scientific evidence then weaved with personal values and experience.

### Policy implications

The objective use of evidence in donor-dependent countries remains a challenge, and part of the solution lies in having strong MoH structures to coordinate prioritized research agendas and KT processes. Although the generation of comprehensive evidence takes time, it remains the best option in order to assess all aspects of policy implications and the feasibility of implementation. Such efforts will need to be planned prior to embarking on the policymaking process. In addition, reaching a consensus on available evidence and the possible course of action is crucial, so as to harnessed to all available resources to facilitate implementation.

### Research implications

This study has also highlighted other factors which would require more exploration to assess whether outcomes would be different if they were in place, in reference to policies categorized as high issue polarization. These included effective and well-coordinated dissemination of evidence, stakeholders reaching a consensus on the available evidence, the MoH having the negotiating power to take a preferred course of action in line with evidence and, reducing the turnover of senior officers to ensure continuity. In addition, testing our MRT on additional case study will lead to more refinement and perhaps improve the extent to which the MRT can be generalized.

## Conclusion

Evidence was used in several ways in the case of the abolition of user fees. We noted symbolic use of evidence in the form of community complaints by the president to abolish user fees; instrumental use of evidence in the form of routine monitoring data by technocrats in the MoH to negotiate for an increase in the health sector allocation and, conceptual use of evidence in the form of survey data by the cabinet to debate the ills of user fees for health care. What is taken as evidence may vary from case to case due to the stakeholders involved and how much interest and influence they wield. We found divergences and convergence with facilitatory factors identified in our earlier MRT on KT in Uganda. Additional factors and themes emerged as well. Respondents perceived the availability of evidence including its dissemination; the MoH institutional capacity to lead KT processes; the existence of partnerships for KT; a favorable political context; and how evidence aligns with the overall government policy discourse as the factors that impacted the uptake of evidence. We refined our initial MRT as “**“**evidence will be taken up in policies so as to lead to evidence informed policies in instances where the MoH leads the KT process, there are partnerships for KT in place and, the nature of the policy issue under consideration, the overall government agenda and; the political situation can be expected to play a role**”** We acknowledge that context is very important in KT and our refined MRT may not hold true in all contexts. Thus, applying the MRT calls for understanding the context within which KT processes occur; the stakeholders involved and; the nature of the policy that is to be influenced by the evidence. However, we believe that our theory can serve as a starting point for other countries planning to abolish user fees for health care and seeking to maximize the use of evidence.

## Additional files

Additional file 1
**Guideline for document review.** Description: Guide used in reviewing documents. Additional file 2
**Document reviewed.** Description: Details of documents reviewed. Additional file 3
**In-depth interview guide for policy makers and researchers.** Description: Open ended questions to elicit responses from respondents.

## Abbreviations

CSOs: Civil society organizations
HSSP: Health Sector Strategic Plan
KI: Key informants
KT: Knowledge translation
MOH: Ministry of Finance
MoH: Ministry of Health
MRT: Middle Range Theory
PEAP: Poverty Eradication Action Plan
SWAp: Sector Wide Approach
UNICEF: United Nations Children Fund
UPPAP: Uganda Poverty Participatory Assessment Project
WB: World Bank
WHO: World Health Organization
