# Supplementary material for: Uptake of evidence in policy development: the case of user fees for health care in public health facilities in Uganda
Source: BMC Health Serv Res. 2014 Dec 18;14:639. doi: 10.1186/s12913-014-0639-5 (PMC4310169; doi:10.1186/s12913-014-0639-5)
Supplement: Additional file 2 — Document reviewed. Description: Details of documents reviewed. [file 12913_2014_639_MOESM2_ESM.doc]

**Table c: Documents and research reports reviewed**

| **Year of development** | **Document** |  |
| --- | --- | --- |
| April 2000 | Discussions of the Budget framework paper for 2000/01 |  |
| June 2000 | Pay Reform in the Uganda health sector over the last 2 decades |  |
| July 2000 | National user charges policy |  |
| July 2000 | Briefing paper on user fees charges for health services |  |
| July 2001 | Summary of discussions at HPAC concerning user charges for health services |  |
| March 2001 | Briefing paper on user fees charges for health services |  |
| February 2002 | Budget frame work paper for the health sector |  |
| March 2002 | Health financing strategy for Uganda |  |
|  |  |  |
| **Research studies reviewed** | |  |
| **Report** | **Findings** | **Recommendations** |
| Kipp W, Kamugisha J, Burnham G, Rubaale T: **Cost-sharing in Kabarole district, Western Uganda: Communities' and Health professionals' perceptions about health financing**. *Journal of Health & Population in Developing Countries* 1999, **2**(2):30 - 38. | Cost sharing project was successful. Enhanced community participation, local decision making and management of funds | Cost sharing has the potential to improve health service delivery but funds should be retained at the local level and spending decisions should also be made at this level. |
| Kipp W, Kamugisha J, Jacobs P, Burnham G, Rubaale T: **User fees, health staff incentives, and service utilization in Kabarole District, Uganda**. *Bull World Health Organ* 2001, **79**(11):1032-1037. | After the introduction of cost sharing, overall utilization of general outpatient services dropped by 21.3%. Utilization increased, however, in facilities  located in remote areas, while it decreased in those located in urban or semi-urban areas. The increased utilization  in remote facilities was considered to be largely attributable to health workers’ incentive payments derived from  cost-sharing revenues. | Ccost sharing may increase the  utilization of health services inspecial circum-  stances. Further research is needed in  order to define these circumstances more precisely  and explore the differences between the rural and  urban situations |
| June 1999 | Inter-Ministerial review of user charges | Maintain user fees where possible but include exemption mechanisms |
| WHO/MoH 3 year longitudinal study on abolition of user fees; 2001 - 2003 | Increased utilization rates for curative services more so among the poor socioeconomic groups, no increases noted in the uptake of preventive services. Increased use of the private sector as well. Reduced staff morale and drug stock outs | Need for system wide improvements to sustained increases in health services utilization |
| Deninger Mpuga & Johannes Kepler University, Linz; 2002 | The improved access to services was, however, not associated with improved outcomes, suggesting that better access was not accompanied by improvements in the quality of services, as is indeed supported by qualitative evidence and the fact that wealthy households seem to have opted out of public services | Need to improve the quality of health services |
| Pariyo et al, (2004) Discontinuation of user fees in Uganda | Increases in uptake of health services for al socio economic categories | Gaps in service deivery affecting uptake of services (drug stock outs, staffing gaps, lack of basic inputs) |
| Xu, K., Evans, D. B., Kadama, P., Nabyonga, J., Ogwal, P. O., Nabukhonzo, P., & Aguilar, A. M. (2006). Understanding the impact of eliminating user fees: utilization and catastrophic health expenditures in Uganda. *Social Science & Medicine*, *62*(4), 866-876. | Utilization increased for the non-poor. Utilization among the poor increased much more rapidly after the abolition of fees than beforehand. The incidence of catastrophic health expenditure among the poor did not fall. The most likely explanation is that frequent unavailability of drugs at government facilities after 2001 forced patients to purchase from private pharmacies. Informal payments to health workers may also have increased to offset the lost revenue from fees | Countries thinking of removing user charges should first examine what types of activities and inputs at the facility level are funded from the revenue collected by fees, and then develop mechanisms to ensure that these activities can be sustained subsequently. |
| Nabyonga, J., Desmet, M., Karamagi, H., Kadama, P. Y., Omaswa, F. G., & Walker, O. (2005). Abolition of cost-sharing is pro-poor: evidence from Uganda. *Health policy and planning*, *20*(2), 100-108. | Marked increase in utilization in all population groups that was fluctuating in nature. Increase in utilization among the poor was more than for other socio-economic categories. Women utilized health services more than men both before and after cost-sharing. Higher increases in utilization were noted among the over-five age group compared with the under-fives. There were no increases in utilization for preventive and inpatient services. | There is a financial barrier created by cost-sharing that decreases access to services, especially among the poor in Uganda. However, further studies are needed to clarify issues of utilization by age and gender |
